# Phenotypic developmental plasticity induced by preincubation egg storage in chicken embryos (*Gallus gallus domesticus*)

**DOI:** 10.14814/phy2.12712

**Published:** 2016-02-23

**Authors:** Sylvia R. Branum, Hiroshi Tazawa, Warren W. Burggren

**Affiliations:** ^1^ Developmental Integrative Biology Research GroupDepartment of Biological SciencesUniversity of North TexasDentonTexas

**Keywords:** Acid–base balance, chicken embryos, fetal programming, hematology, preincubation egg storage, viability

## Abstract

The developing chicken blastoderm can be temporarily maintained in dormancy below physiological zero temperature. However, prolonged preincubation egg storage impairs normal morphological and physiological development of embryos in a potential example of fetal programming (in this case, “embryonic programming”). We investigated how preincubation egg storage conditions (temperature, duration, hypoxia, and hypercapnia) affects viability, body mass, and physiological variables and functions in day 15 chicken embryos. Embryo viability was impaired in eggs stored for 2 and 3 weeks, with the effects greater at 22°C compared to 15°C. However, embryo size was reduced in eggs stored at 15°C compared with 22°C. Phenotypic change resulting from embryonic programming was evident in the fact that preincubation storage at 15°C diminished hematocrit (Hct), red blood cell concentration ([RBC]), and hemoglobin concentration ([Hb]). Storage duration at 15°C more severely affected the time course (2, 6, and 24 h) responses of Hct, [RBC], and [Hb] to progressive hypoxia and hypercapnia induced by submersion compared with storage duration at 22°C. The time‐specific regulation of acid–base balance was changed progressively with storage duration at both 22 and 15°C preincubation storages. Consequently, preincubation egg storage at 22°C resulted in poor viability compared with eggs stored at 15°C, but size and physiological functions of embryos in eggs stored for 1–2 weeks were worse in eggs stored in the cooler than stored under room conditions. Avian eggs thus prove to be useful for examining developmental consequences to physiology of altered preincubation thermal environment in very early stages of development (embryonic programming).

## Introduction

Fetal programming occurs when experiences during human fetal development cause phenotypic changes later in life. This phenomenon in humans has rightfully received much attention due to the potential effects of fetal experiences on health in late adulthood (see Alexander et al. 2015; Barker 2002; Barker et al. 2002; Moritz et al. 1984; Roberts et al. 2015; Sedaghat et al. 2015; Stangenberg et al. 2015; for an entry into the voluminous literature). Much less appreciated or even understood, however, is what might be called “embryonic programming,” in which experiences of the embryo, beginning with fertilization of an egg by a sperm, have downstream consequences to phenotype of the fetus and ultimately the adult. As one example, evidence of the effects of environment on the very earliest stages of development after fertilization are emerging from studies on in vitro fertilization procedures used in human and domestic mammals. Variations in the environment produced by the culture medium in which the newly fertilized human eggs are cultured for the first 5 days have been reported to alter birth weight and gender ratio, and can otherwise be detrimental (Vergouw et al. 2012; Kleijkers et al. 2015; Zhu et al. 2015). However, the influence of culture medium on embryonic and subsequent fetal and neonatal phenotype remains controversial, with alternative findings of no effect of culture medium (Lin et al. 2013; De Vos et al. 2015). Certainly in mammalian models, however, maternal dietary protein alters the periconception environment, with developmental plasticity subsequently evident in embryonic development (Eckert et al. 2012; Fleming et al. 2015).

Embryonic programming is likely a process basic to all vertebrates, given the fundamental nature of the process of embryonic fertilization and early embryonic growth. Embryonic programming is certainly evident in avian eggs, where a number of conditions including the endocrine messages loaded into the egg at laying, chronic moderate hypoxia during incubation, or embryonic undernutrition exert influence on the developing embryo with cardiovascular and renal functions, even at early stages and in adults (Ruijtenbeek et al. 2003a,b; Nishimura et al. 2007; Nelson et al. 2010; Read and Clark 2011; Adkins‐Regan et al. 2013). Even the act of storage below so‐called “physiological zero” of newly fertilized bird eggs pending incubation and development can have subsequent developmental implications. In chicken eggs, for example, there is a time limit for successful storage that retains full hatchability – typically about 1 week (Haque et al. 1996; Lapão et al. 1999; Fasenko 2007; Reijrink et al. 2010; Hamidu et al. 2011). Prolonged preincubation egg storage causes regressive changes in blastoderm morphology, retarded growth, malformation and mortality of embryos, decreased hatchability, and increased incubation period (Arora and Kosin 1966; Mather and Laughlin 1976, 1977; Haque et al. 1996; Fasenko 2007). Prolonged egg storage, especially when combined with temperature fluctuations, also adversely affects albumen quality, an indicator of overall egg quality (Hurnik et al. 1978; Scott and Silversides 2000; Reyna 2010; Reyna and Burggren 2012). In addition, physiological functions of these embryos are inhibited (Haque et al. 1996; Christensen et al. 2001; Fasenko 2007). For example, the O_2_ consumption rates of embryos developing in eggs that were stored for 20 and 30 days at 10–11°C prior to incubation are depressed, and the developmental trajectories for changes in heart rate are flattened compared with those of control eggs. As a result, O_2_ consumption with every heartbeat, that is, O_2_ pulse, is markedly lowered by prolonged preincubation egg storage, decreasing blood O_2_ transport, and inhibiting embryo growth (Haque et al. 1996).

Storage length and its potential effects on bird embryos and their ultimate phenotype can inform us about embryonic programming, but there are additional practical implications of such studies. Because of these undesirable disadvantages of preincubation egg storage, it is a common procedure in the poultry breeding industry to limit cool egg storage to less than 1 week. Thus, laboratory researchers, vaccine producers, etc., are typically provided from the breeders with preincubation eggs less than 1‐week‐old. After arrival at a laboratory, however, eggs are often additionally stored at or below room temperature until incubation begins according to an experimental schedule. Since laboratory room temperature is typically maintained below physiological zero (the temperature required to initiate blastoderm development or maintain dormancy of embryos), eggs can be kept at room temperature for a few days before incubation.

Despite indications of storage effects on the embryos (embryonic programming), we know little of the physiological effects of variable storage conditions and length on the physiology of the embryo, and thus on the ultimate phenotype of the developing bird. Consequently, in this study we have determined the effect of fertilized egg storage as a function of time and temperature on the embryo viability, size, and physiological functions (hematological respiratory variables and acid–base disturbances during progressive intrinsic hypercapnia and hypoxia) as evident on day 15 (d15) of development in the embryos of broiler chickens. We hypothesize that duration and temperature of preincubation egg storage will not only affect embryo viability, but also alter hematocrit regulation and acid–base metabolic compensation, reflecting embryonic programming of physiological phenotype.

## Materials and Methods

### Preincubation egg storage, water loss from eggs, and viability of embryos

All chicken embryo experiments were conducted at the University of North Texas in accordance with the protocol approved by the UNT Institutional Animal Care and Use Committee. Preincubation egg storage experiments were conducted on six replicates with a few indicated modifications among them (Table 1). Fertile eggs of broiler (Cornish Rock) chickens were supplied 12 times from a local hatchery. On each arrival at the laboratory, eggs were cleaned lightly with water and a sponge to eliminate material covering the eggshell and possibly interrupting gas exchange. After drying, 120 eggs comprising half of a single replicate according to storage temperature (~22 or 15°C) were weighed with an electronic balance to 0.01 g and randomly divided into four groups according to storage duration; nonstorage control group (hereafter, conveniently referred to as 0 week), 1, 2, and 3 weeks storage groups. Each storage group consisted of 30 eggs and eggs in the 0 week control group were placed at noon into an incubator warmed at a temperature of 37.5°C and ~60% of relative humidity (RH). Eggs of other storage groups were stored on a bench either in the laboratory or in a cold room for 1, 2, or 3 weeks before start of incubation at noon. Laboratory temperature was ~22°C on average ranging from ~20 to 24°C. RH varied between ~25% and 50%, mainly ~35–40%. The temperature and RH of the cold room were regulated at 15°C and 50%, respectively. On the day of start of incubation after the elapse of the designated storage durations, mass of each stored egg was measured to 0.01 g. The difference of egg mass between on the day of arrival and start of incubation was determined as water loss from the egg during storage as an alternative indicator of RH. In summary, six replicates, each replicate comprising 0, 1, 2, or 3 weeks storage groups, were examined for effects of storage at ~22°C or at 15°C. The 22°C storage group in the sixth replicate was stored in a box with a RH of ~80% to reduce water loss and compare effects of water loss across groups.

**Table 1:** The date of eggs’ arrival at the laboratory in six replicates of egg storage procedures at 22 and 15°C with hens’ age (week, when available) and mean egg mass (g, with SEM, *N* = 120) at arrival. The different symbol on egg mass indicates significant difference among six mean values at 22 or 15°C storage procedure

Replicate No.	Storage temperature					
	22°C			15°C		
	Arrival (m/d/y)	Hens’ age	Egg mass	Arrival (m/d/y)	Hens’ age	Egg mass
1	4/11/13	–	65.70 ± 0.32^a^ (*N* = 120)	5/08/13	–	65.15 ± 0.34^a^ (120)
2	5/22/13	–	59.19 ± 0.34^b^ (120)	9/29/13	–	63.38 ± 0.35^ab^ (120)
3	10/25/13	–	65.16 ± 0.30^a^ (120)	11/07/13	–	65.10 ± 0.32^a^ (120)
4^a^	3/02/14	52	65.22 ± 0.34^a^ (120)	3/04/14	52	65.59 ± 0.30^a^ (120)
5^b^	4/11/14	27	51.40 ± 0.29^c^ (120)	4/09/14	27	51.55 ± 0.32^c^ (120)
6^b^	5/07/14	31	59.29 ± 0.23^bc^ (120)	5/09/14	31	59.77 ± 0.27^bc^ (120)
Six replicates			61.00 ± 0.27 (*N* = 720)			61.75 ± 0.23 (*N* = 720)

The eggs of replicate no. 6 in 22°C storage procedure were stored in a box maintained at ~80% of RH.

a Sibling eggs laid by hens of flock #1.

b Sibling eggs laid by hens of flock #2.

On d14 of incubation, 30 eggs in each of 0, 1, 2, and 3 weeks storage groups stored at ~22 or 15°C were candled to examine if embryos were developing and the eggs containing embryos were counted as viable eggs (Table 2). The viability of embryos was determined as percent ratio of the number of viable eggs (embryos) to 30 eggs.

**Table 2:** Number of viable embryos among 30 eggs and viability (%, in parentheses) at 0 (nonstorage), 1, 2, and 3 weeks of 22 or 15°C storage in six replicates of storage procedure. Viability of embryos in each replicate of storage procedure is a percent ratio of number of viable embryos to 30 eggs and mean viability is the value averaged for individual viability in six replicates of storage procedure

Replicate No.	Storage temperature							
	22°C				15°C			
	Storage duration				Storage duration			
	0 week	1 week	2 weeks	3 weeks	0 week	1 week	2 weeks	3 weeks
1	24 (80.0)	24 (80.0)	16 (53.3)	0 (0)	28 (93.3)	28 (93.3)	20 (66.7)	4 (13.3)
2	26 (86.7)	22 (73.3)	17 (56.7)	1 (3.3)	25 (83.3)	26 (86.7)	24 (80.0)	17 (56.7)
3	28 (93.3)	24 (80.0	7 (23.3)	0 (0)	30 (100)	25 (83.3)	15 (50.0)	12 (40.0)
4^a^	26 (86.7)	21 (70.0)	2 (6.7)	0 (0)	24 (80.0)	22 (73.3)	16 (53.3)	7 (23.3)
5^b^	26 (86.7)	21 (70.0)	2 (6.7)	0 (0)	24 (80.0)	24 (80.0)	21 (70.0)	10 (33.3)
6^b^	28 (93.3)	29 (96.7)	4 (13.3)	0 (0)	28 (93.3)	24 (80.0)	20 (66.7)	12 (40.0)
Total	158	141	48	1	159	149	116	62
Mean viability (%)	87.8	78.3	26.7	0.6	88.3	82.8	64.5	34.4

a Sibling eggs laid by hens of flock #1.

b Sibling eggs laid by hens of flock #2.

The eggs containing viable embryos were lined in red on their equator for the partial water submersion experiment. Additionally, the eggshell over the allantoic vein was marked for blood collection on d15.

### Progressive hypercapnia and hypoxia by water submersion

Partial water submersion of the egg has been established as a technique for testing physiological regulation in chicken embryos (Andrewartha et al. 2014). The viable eggs from the control (without storage) and the individual storage groups described above were divided into four subgroups: 0 (control without submersion), 2, 6, and 24 h of submersion (Table 3). Eggs were transferred into a desk‐top incubator warmed to 37.5°C and provided with a vat containing water and a plastic egg stand on d14. The eggs were first placed on a cardboard egg stand with air cell down. The first subgroup was deemed the control (0 h). The two other subgroups were submerged in water for 2 and 6 h on d15. The fourth subgroup was submerged on d14 for 24 h of submersion. The eggs were placed on the plastic stand in the vat, air cell down, and submerged in water up to the red line on the equator. Among viable embryos, a few embryos died during 24 h of submersion and blood collection failed in several other embryos. These embryos were not included in the result of the water submersion experiment (Table 3).

**Table 3:** Number of embryos successfully subjected to water submersion experiment on days 14–15 of incubation

Storage temperature (°C)	Storage duration (week)	Submersion time				Total
		0 h	2 h	6 h	24 h	
22	0	38	38	38	36	150
	1	34	34	34	34	136
	2	10	12	12	9	43
	3	1	0	0	0	1
15	0	37	37	37	38	149
	1	34	35	36	35	140
	2	26	26	26	27	105
	3	16	16	16	16	64

### Blood collection and analysis

After the designated time of submersion had elapsed, the previously submerged half of the egg was covered with Parafilm^®^ to preserve blood gas levels during blood collection (Burggren et al. 2012). A window ~1 cm across was torn in Parafilm^®^ over the site of the allantoic vein and a small hole ~0.8 cm across opened in the eggshell. For control eggs, the hole was directly opened in the eggshell. Approximately 0.4 mL of blood was immediately collected from the allantoic vein into a 1 mL heparinized plastic syringe. Blood was gently emptied into a 1.5‐mL plastic vial and immediately analyzed for pH, [HCO_3_^−^] (mmol L^−1^) and pco_2_ (mmHg) by a blood gas system (ABL5, Radiometer Medical A/S, Denmark).

The remaining blood was well stirred in the vial and measured for red blood cell concentration ([RBC], 10^6^ cells *μ*L^−1^) and hemoglobin concentration ([Hb], g%) using a hematology analyzer (Beckman Coulter Analyzer A^c^T10) and hematocrit (Hct, %) with a centrifuge (Readacrit Centrifuge, Becton Dickinson). Hct was determined on duplication samples and two determinations were averaged for a value of Hct in individual embryos. [RBC] determined by the Coulter Analyzer was modified using an expression previously derived from a relationship with [RBC] determined by a hematometer (Tazawa et al. 2011). Mean corpuscular volume (MCV, *μ*m^3^), mean corpuscular hemoglobin (MCH, pg), and mean corpuscular hemoglobin concentration ([MCHb], g%) were calculated from Hct, [RBC], and [Hb] (i.e., MCV = 10·Hct/[RBC], MCH = 10·[Hb]/[RBC], and [MCHb] = 100·[Hb]/Hct). Additionally, blood osmolality (Osm, mmol kg^−1^) was measured with a vapor pressure osmometer (5520 Vapro, Wescor Inc.) and lactate concentration ([La^−^], mmol L^−1^), with a lactate meter (Nova Lactate Plus Meter, Nova Biomedical, MA).

### Embryonic body mass

After blood collection, embryos were euthanized by putting the eggs in a plastic bag ventilated by N_2_. The yolk and extraembryonic membranes were removed and excess water around the body of embryos was removed by blotting the embryos repeatedly on a paper. The wet body mass (BM) of the embryos was determined to 0.01 g with the electronic balance.

### Statistical analysis

All data were tested for normality and equal variance and parametric ANOVA or ANOVA on ranks was used where appropriate. The correlations between viability and BM of embryos and between storage duration and water loss from the eggs were examined for the significance by *t* test. Comparison between two mean values of egg mass in individual groups of 22 and 15°C storage procedures and comparison between mean viability of embryos between 22 and 15°C storage procedures at each storage week were made by unpaired Student's *t* test. Differences in mean values of egg mass across the six replicates of 22 and 15°C storage procedures were examined using a one‐way ANOVA with all pairwise multiple comparisons by the Tukey's test or Dunn's method. The effects of storage temperature and storage duration on viability and BM of embryos were examined for their significance by a two‐way ANOVA with all pairwise multiple comparison procedures by Holm–Sidak method. The effects of storage temperature, storage duration, and submersion time on BM, Osm, [La^−^], and hematological respiratory variables (Hct, [RBC], MCV, [Hb], MCH, and [MCHb]) were examined for their significance by a three‐way ANOVA. In time‐specific responses to submersion, differences in mean values of variables (BM, Osm, [La^−^], and hematological respiratory variables) between all storage procedures (0, 1, and 2 weeks at 22°C and 0, 1, 2, and 3 weeks at 15°C) at each submersion time were examined using a one‐way ANOVA. Differences in mean values of blood gas variables (pH, [HCO_3_^−^], pco_2_) between storage groups at all storage times were examined by a one‐way ANOVA. The significance level was set at *P* < 0.05 for all tests. All data were presented as mean ± 1 SEM.

## Results

### Viability and body mass of embryos and water loss from eggs during storage

In the first three replicates individually comprising 22 and 15°C storage procedures, experiments with the 22°C storage group was first undertaken and subsequently after completion the experiments with the 15°C group was made (Table 1). The remaining three replicates were carried out in parallel with a 2‐day lag between 22 and 15°C storage procedures. Replicate 4 comprised sibling eggs laid by 52 weeks old hens. The eggs of replicates 5 and 6 were siblings laid by hens of another flock when they were 27 and 31 weeks old. The eggs of replicate 5 were the smallest and ~1 month later egg mass increased, but they were still smaller than the eggs in the other replicates. Consequently, the mean egg mass among six replicates of 22 or 15°C storage procedures was significantly different (*P* < 0.001).

**Figure 1:**
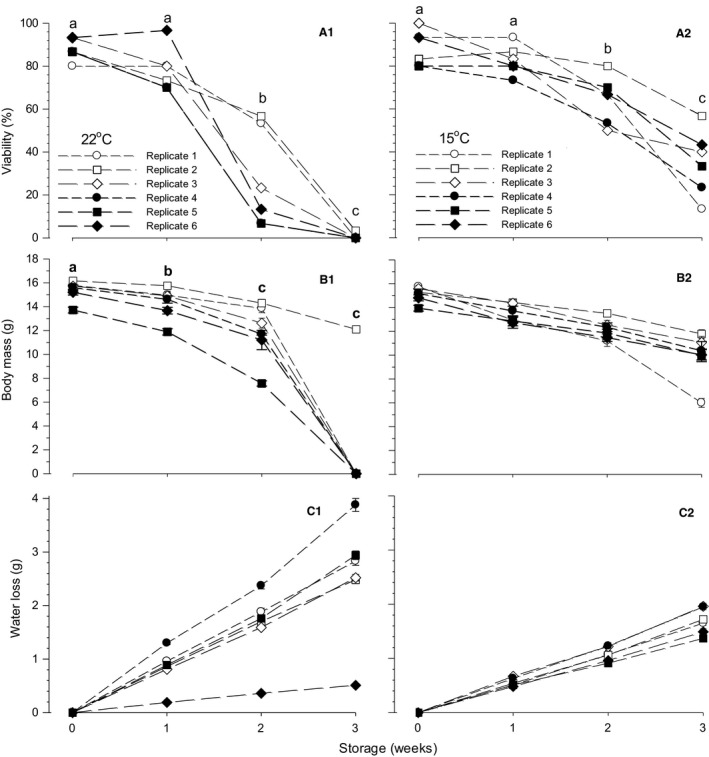
Changes in viability (panel A1, A2), body mass (panel B1, B2), and egg water loss (panel C1, C2) in d15 embryos stored as eggs for 3 weeks at 22 and 15°C in six replicates of storage procedure. Viability of embryos in each group of 22 and 15°C storage procedures is percent ratio of number of viable embryos (shown in Table 2) to 30 eggs. Body mass is mean value of viable embryos and water loss is mean value of 30 eggs. The different symbols in panels A1, B1, and A2 indicate significant difference between mean values of storage durations (0 (nonstorage), 1, 2, and 3 weeks). When the interaction between storage temperature and storage duration was not significant, the significance symbols are shown only in a 22°C panel. Mean values ± 1 SEM are plotted in panels B1, B2, C1, and C2.

Embryo viability was significantly affected by storage temperature (*P* < 0.001) and storage duration (*P* < 0.001) and their interaction (*P* < 0.001) (Fig. 1A1, A2; Table 2). For storage at 22 and 15°C, the mean viability was 48.3% and 67.6%, respectively. For storage duration, the mean viability was 88.1%, 80.6%, 45.6%, and 17.8% for 0, 1, 2, and 3 weeks, respectively. The difference of mean viability between 22 and 15°C storage procedures was not significant at 0 week (87.8 cf. 88.3%, *P* = 0.938) and 1 week (78.3 cf. 82.8%, *P* = 0.521), but became significant at 2 weeks (26.7 cf. 64.5%, *P* < 0.001) and 3 weeks of storage (0.6 cf. 34.4%, *P* < 0.001). Overall, the viability in the individual six replicates of 22 and 15°C storage procedures was kept high even after 1 week storage that was comparable to 0 week. However, the viability significantly decreased after 2 and 3 weeks of storage. Particularly in 22°C storage procedure, there was a large decrease in viability after 2 weeks and only a single embryo among 180 eggs was viable after 3 weeks of storage.

Body mass of d15 embryos was significantly affected by storage temperature (*P* = 0.033) and storage duration (*P* < 0.001) without significant interaction (*P* = 0.217) (Fig. 1B1, B2). The mean BM in 22°C storage (13.77 ± 0.45 g, *N* = 348) was significantly higher compared with that in 15°C storage (12.80 ± 0.08 g, *N* = 486). The mean BM significantly decreased with storage duration, that is, 15.25 ± 0.10 (*N* = 317), 13.90 ± 0.10 (290), 12.71 ± 0.15 (164), and 11.29 ± 0.89 (63) g for 0, 1, 2, and 3 weeks storage. The BM shown by the filled square symbols in 22°C storage corresponded to embryos in replicate 5 with the lowest egg mass (Fig. 1B1).

Water loss from eggs during storage increased linearly with storage duration in both 22 and 15°C storage with significant coefficients of correlation between water loss and storage duration (Fig. 1C1, C2). The mean correlation of replicates 1–5 (except 6) in 22°C storage was 0.97 ± 0.08 g wk^−1^, which was larger than 15°C storage (0.56 ± 0.03 g wk^−1^) (*P* < 0.001). Correlation of replicate 6 in 22°C storage, of which water loss was reduced by storing eggs in ~80% RH environment, was 0.17 g wk^−1^ (diamond symbols in Fig. 1C1).

**Figure 2:**
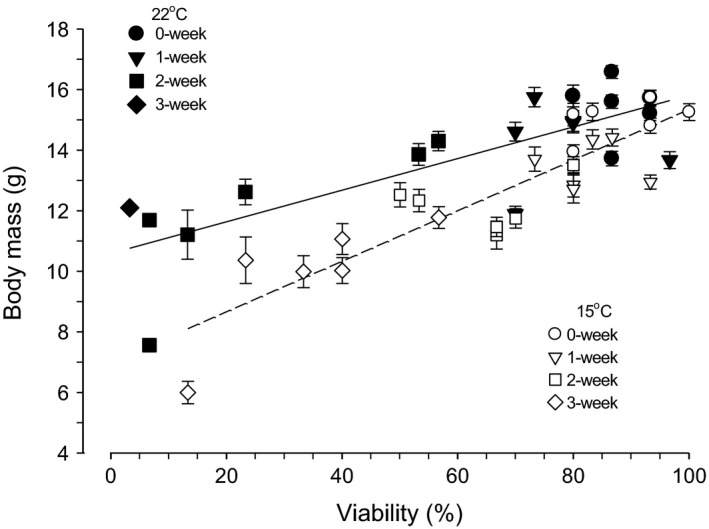
The relation between viability and body mass in d15 embryos stored as eggs at 22 and 15°C for 0 (nonstorage), 1, 2, and 3 weeks. Individual values of viability and body mass are the same as those in Figure 1.

The viability and BM, which decreased with storage duration in both 22 and 15°C storage procedures (Fig. 1A1, B1, A2, B2), had a significant relation between decreases in both variables (Fig. 2); correlation was 0.052 (*r* = 0.791, *t* = 5.336, *P* < 0.001) and 0.084 g/% (*r* = 0.887, *t* = 8.999, *P* < 0.001) in 22 and 15°C storage, respectively.

### Responses of physiological variables and functions to submersion

#### Body mass, osmolality, and lactate concentration

**Figure 3:**
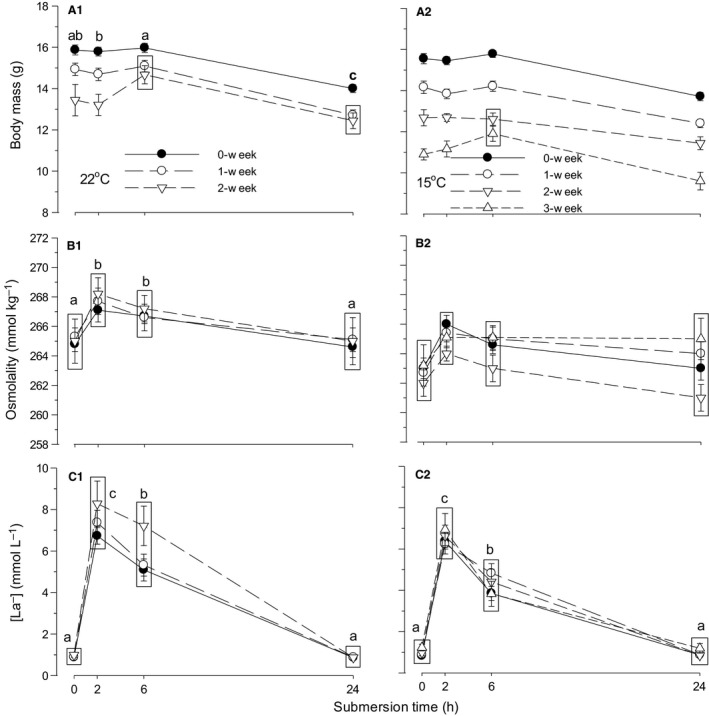
Time‐specific changes in body mass (panel A1, A2), blood osmolality (B1, B2), and lactate concentration ([La^−^]) (C1, C2) during 24 h of water submersion in d15 embryos stored as eggs for 0 (nonstorage), 1, and 2 weeks at 22°C and additionally 3 weeks at 15°C. Mean values ± 1 SEM are plotted (*N* is shown in Table 3). Means not significantly different from each other are grouped within the same box. Different letters indicate significant difference between submersion times for all storage procedures (except 3 weeks) combined. When the interaction between storage temperature and submersion time was not significant, the significance letters are shown only in a 22°C panel.

Because only a single embryo was viable among eggs stored for 3 weeks at 22°C, the results obtained from this embryo and eggs stored for 3 weeks at 15°C were not included for comparison between effects of storage temperature, storage duration, and submersion time on BM, Osm, and [La^−^] (Fig. 3).

The effects of storage temperature, storage duration, and submersion time on BM at d15 were all significant with only significant interaction between storage temperature and storage duration (Table 4). The overall BM of embryos in eggs stored at 22°C (14.40 ± 0.10 g, *N* = 329) was significantly heavier than that in eggs stored at 15°C (13.71 ± 0.07 g, *N* = 394) (Fig. 3A1, A2). In 0 week storage (nonstorage control group), the combined BM was not different between 22 (15.41 ± 0.12 g, *N* = 150) and 15°C (15.12 ± 0.12 g, *N* = 149) groups (*P* = 0.091), but BM changed between 22 and 15°C in 1 week and 2 weeks storages with heavier BM at 22°C than 15°C (*P* < 0.001 for both storages). The BM was significantly deteriorated by extension of storage duration in both 22 and 15°C egg storage groups, that is, combined BM was 15.27 ± 0.08 (*N* = 299), 14.00 ± 0.09 (276), and 12.90 ± 0.14 (148) g for 0, 1, and 2 weeks, respectively (*P* < 0.001 for all three comparisons). The combined BM of 22 and 15°C storage eggs became significantly smaller in embryos of eggs submerged for 24 h compared with 0, 2, and 6 h submersion (*P* < 0.001 for all three comparisons).

**Table 4:** Results from a three‐way ANOVA and Holm–Sidak test for the effects of preincubation storage temperature (Temp), preincubation storage duration (Duration), and submersion time (Time) and the interactions between the three treatments (Temp × Duration, Temp × Time, Duration × Time, Temp × Duration × Time) on body mass (BM), osmolality (Osm), lactate concentration ([La^−^]), and hematological respiratory variables (Hct, [RBC], MCV, [Hb], MCH, and [MCHb]) in day 15 chicken embryos

	Treatments			Interactions			
	Temp	Duration	Time	Temp × Duration	Temp × Time	Duration × Time	Temp × Duration × Time
BM	<0.001	<0.001	<0.001	0.031	0.386	0.583	0.465
Osm	<0.001	0.197	<0.001	0.065	0.959	0.995	0.959
[La^−^]	<0.001	0.028	<0.001	0.230	0.002	0.371	0.393
Hct	<0.001	<0.001	<0.001	0.013	0.314	0.059	0.781
[RBC]	<0.001	<0.001	<0.001	0.012	0.675	0.591	0.903
MCV	0.333	0.077	<0.001	0.402	0.442	0.006	0.692
[Hb]	0.041	<0.001	<0.001	0.047	0.380	0.855	0.915
MCH	<0.001	0.477	<0.001	0.286	0.961	0.675	0.521
[MCHb]	<0.001	0.571	<0.001	0.798	0.397	0.006	0.990

BM, Body mass (g); Osm, Osmolality (mmol kg^−1^); [La^−^], Lactate concentration (mmol L^−1^); Hct, Hematocrit (%); [RBC], Red blood cell concentration (10^6^ *μ*L^−1^); MCV, Mean corpuscular volume (*μ*m^3^); [Hb], Hemoglobin concentration (g%); MCH, Mean corpuscular hemoglobin (pg); [MCHb], Mean corpuscular hemoglobin concentration (g%).

The effects of storage temperature and submersion time on blood Osm were significant, but neither the effect of storage duration nor the interactions were significant (Table 4). The overall Osm of embryos in eggs stored at 22°C (266.1 ± 0.1 mmol kg^−1^) was significantly higher than that in eggs stored at 15°C (263.6 ± 0.2) (Fig. 3B1, B2). The combined Osm of 22 and 15°C storage eggs significantly increased at 2 and 6 h of submersion and subsequently returned to the control levels at 24 h.

The effects of storage temperature, storage duration, and submersion time on [La^−^] were all significant, with only significant interaction between storage temperature and submersion time (Table 4). The overall [La^−^] of embryos in eggs stored at 22°C (3.8 ± 0.1 mmol L^−1^) was significantly larger than that in eggs stored at 15°C (3.1 ± 0.1 mmol L^−1^) (Fig. 3C1, C2). At 0 and 24 h submersion, the combined [La^−^] was not different between embryos in eggs stored at 22 and 15°C (e.g., 0.9 ± 0.3 [*N* = 82] at 22°C cf. 0.9 ± 0.2 [97] mmol L^−1^ at 15°C at 0 h, *P* = 0.950). However, [La^−^] became different between 22 and 15°C at 2 h and 6 h submersions with larger [La^−^] at 22°C than at 15°C. In eggs stored at both 22 and 15°C, [La^−^] rapidly changed with water submersion, being highest at 2 h and decreasing at 6 h, with a subsequent return to control levels at 24 h (Fig. 3C1, C2).

#### Hematological respiratory variables

**Figure 4:**
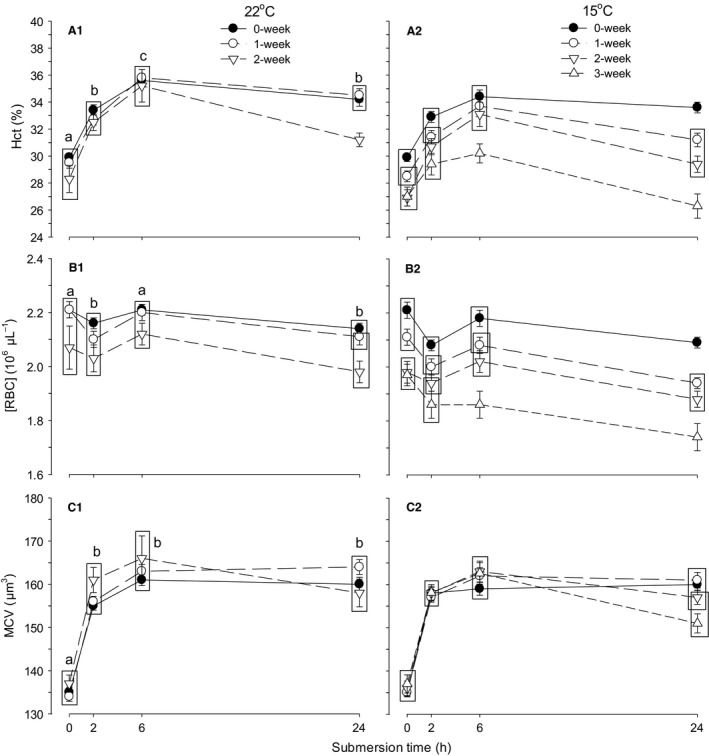
Time‐specific changes in hematocrit (Hct) (panel A1, A2), red blood cell concentration ([RBC]) (B1, B2), and mean corpuscular volume (MCV) (C1, C2) during 24 h of water submersion in d15 embryos stored as eggs for 0 (nonstorage), 1, and 2 weeks at 22°C and additionally 3 weeks at 15°C. Mean values ± 1 SEM are plotted (*N* is shown in Table 3). Means not significantly different from each other are grouped within the same box. Different letters indicate significant difference between submersion times for all storage procedures (except 3 weeks) combined.

The effects of storage temperature, storage duration, and submersion time on Hct and [RBC] were all significant, with only significant interaction between storage temperature and storage duration (Table 4). The overall Hct of embryos in eggs stored at 22°C (32.74 ± 0.19%) was significantly higher than that in eggs stored at 15°C (31.30 ± 0.15%) (Fig. 4A1, A2). In 0 week storage, the combined Hct was not different between 22 and 15°C groups, but Hct became different between 22 and 15°C in 1 week and 2 weeks storages with larger Hct at 22°C than at 15°C (*P* < 0.001 for both storages). Hct was significantly decreased by extended storage duration, that is, significant decrease in Hct occurred in eggs stored for 2 weeks at 22°C compared with 0 (*P* = 0.011) and 1 week (*P* = 0.017) storage eggs and in 15°C storage the decrease in Hct from the control (0 week) was significant even in 1 week storage (*P* < 0.001) with significant decrease from 1 week to 2 weeks storage (*P* = 0.001). In response to submersion, the combined Hct of 22°C and 15°C storage eggs increased at 2 h, peaking at 6 h with subsequent decrease at 24 h to a level at 2 h (28.82 ± 0.24%, 32.27 ± 0.23%, 34.64 ± 0.23%, and 32.34 ± 0.24% at 0, 2, 6, and 24 h, respectively).

The overall [RBC] of embryos in eggs stored at 22°C (2.13 ± 0.01 × 10^6^ *μ*L^−1^) was significantly higher than that in eggs stored at 15°C (2.04 ± 0.01 × 10^6^ *μ*L^−1^), with significant difference in 1 week (*P* < 0.001) and 2 weeks storages with larger [RBC] at 22°C than at 15°C (*P* = 0.001) (Fig. 4B1, B2). The [RBC] was significantly decreased by extended storage, that is, significant decrease in [RBC] occurred in eggs stored for 2 weeks at 22°C compared with 0 and 1 week storage eggs (*P* < 0.001 for both comparisons), while [RBC] significantly decreased with every storage durations from 0 to 2 weeks at 15°C storage (*P* < 0.001 for all comparisons). In response to submersion, the combined [RBC] of 22 and 15°C storage eggs decreased at 2 h and returned to control level at 6 h with subsequent decrease at 24 h.

Only submersion time significantly affected MCV, with the only significant interaction occurring between storage duration and submersion time (Table 4). MCV markedly increased at 2 h of submersion (*P* < 0.001), keeping the same level during the remaining period in nonstorage eggs, increasing at 6 and 24 h in 1 week storage eggs (*P* < 0.001 for both times compared with 2 h) or decreasing at 24 h after increase at 6 h in 2 week storage eggs (*P* = 0.003) (Fig. 4C1, C2).

**Figure 5:**
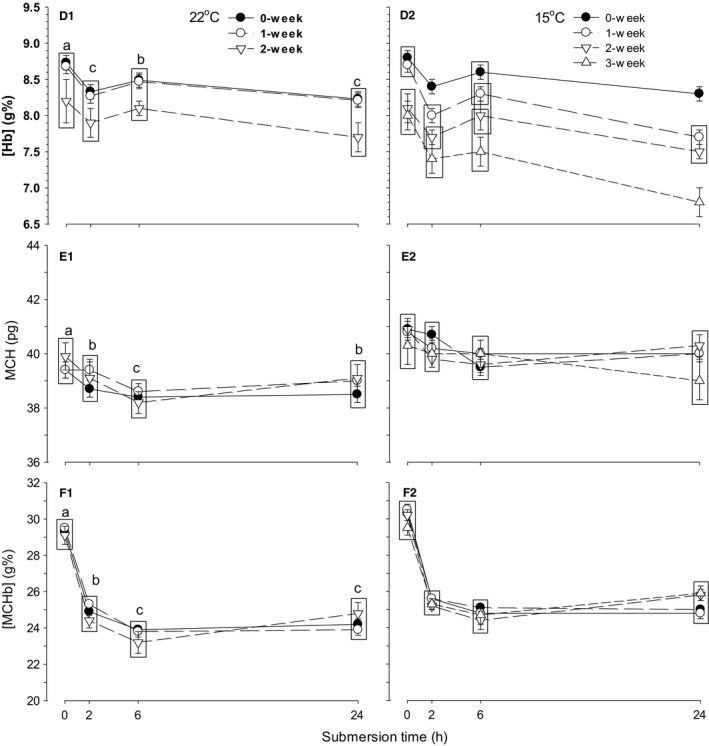
Time‐specific changes in hemoglobin concentration ([Hb]) (panel D1, D2), mean corpuscular hemoglobin (MCH) (E1, E2), and mean corpuscular hemoglobin concentration ([MCHb]) (F1, F2) during 24 h of water submersion in d15 embryos stored as eggs for 0 (nonstorage), 1, and 2 weeks at 22°C and additionally 3 weeks at 15°C. Mean values ± 1 SEM are plotted (*N* is shown in Table 3). Means not significantly different from each other are grouped within the same box. Different letters indicate significant difference between submersion times for all storage procedures (except 3 weeks) combined.

The effects of storage temperature, storage duration, and submersion time on [Hb] were significant with only significant interaction between storage temperature and storage duration (Table 4). The overall [Hb] of embryos in eggs stored at 22°C was significantly lower in eggs stored for 2 weeks compared with 0 and 1 week storage eggs (*P* < 0.001 for both comparisons) (Fig. 5D1). However, in 15°C storage, [Hb] was significantly decreased by each week of storage (*P* < 0.001 for all comparisons) (Fig. 5D2). In response to submersion, the combined [Hb] of 22 and 15°C storage eggs decreased at 2 h and increased at 6 h with subsequent decrease at 24 h. The effects of storage temperature and submersion time on MCH were significant, but the effect of storage duration was not significant, with nonsignificant interactions between all the procedures (Table 4). The overall MCH of embryos in eggs stored at 15°C (40.2 ± 0.1 pg) was significantly higher than that stored at 22°C (39.0 ± 0.1 pg). The combined MCH in eggs stored at 22 and 15°C decreased significantly at 2 h and further at 6 h with subsequent increase to 2 h level after 24 h (Fig. 5E1, E2). The effects of storage temperature and submersion time on [MCHb] were significant, but the effect of storage duration was not significant with only significant interaction between storage duration and submersion time (Table 4). The overall [MCHb] of embryos in eggs stored at 15°C (26.5 ± 0.1 g%) was significantly higher than that stored at 22°C (25.5 ± 0.1). The combined [MCHb] in eggs stored at 22 and 15°C decreased significantly at 2 h and further at 6 and 24 h (*P* < 0.001 for 2, 6, and 24 h from control) (Fig. 5F1, F2).

In 22°C storage procedures, values of Hct, [RBC], MCV, [Hb], MCH, and [MCHb] determined at 0 h were not different between groups of 0, 1, and 2 weeks storage (Fig. 4A1; *P* > 0.2 for all hematological variables). In contrast, in 15°C storage, values of Hct, [RBC], and [Hb] at 0 h were significantly different between 0 and 1 week of storage and 2 and 3 weeks storage procedures (Figs. 4A2, B2, 5D2; *P* < 0.001 for all comparisons) with a significant difference occurring at 2, 6, and 24 h between 0, 1, 2, and 3 weeks storage procedures (*P* < 0.001 for all comparison).

#### Acid–base balance

In a previous study (Burggren et al. 2012), the relationship between blood pH and [HCO_3_^−^] in d15 embryos was depicted on a Davenport (pH‐[HCO_3_^−^]) diagram constructed by plotting pco_2_ isopleths calculated from the Henderson–Hasselbalch equation using a CO_2_ solubility factor of 0.0308 mmol L^−1^ mmHg^−1^ and serum carbonic acid pK’ varying with pH. A buffer line was drawn on the Davenport diagram to indicate the previously determined buffer value of −16 mmol L^−1^ pH^−1^ (Burggren et al. 2012).

**Figure 6:**
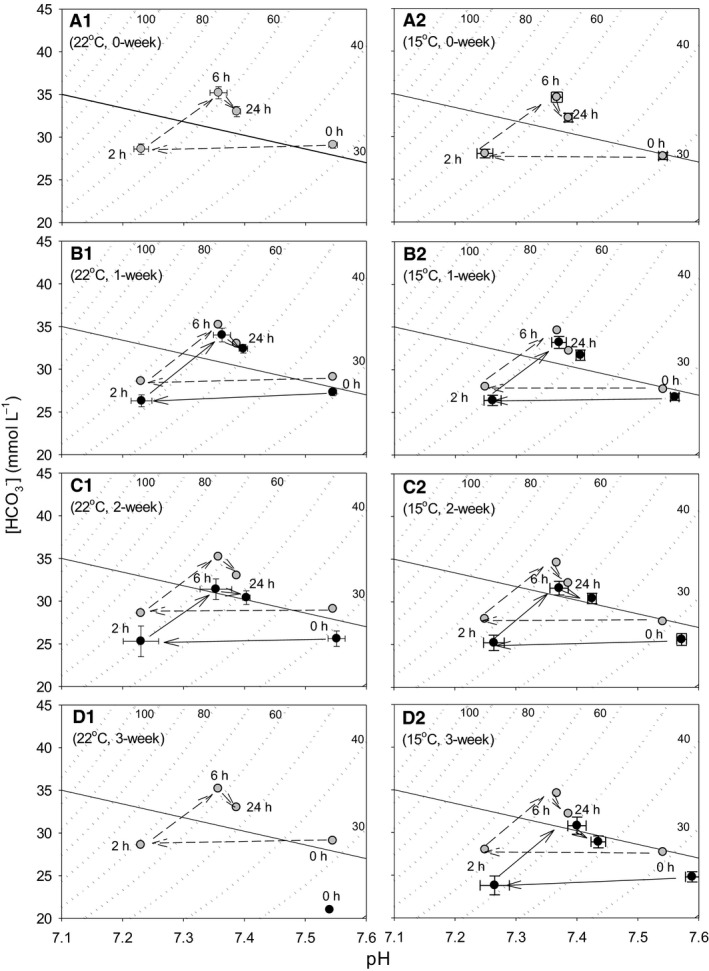
Time‐specific changes of acid–base disturbances during 24 h of water submersion in d15 embryos stored as eggs for 0 (panel A1), 1 (B1), 2 (C1), and 3 weeks (0 h only) (D1) at 22°C, and in eggs stored for 0 (panel A2), 1 (B2), 2 (C2), and 3 (D2) weeks at 15°C. Mean values ± 1 SEM are plotted (*N* is shown in Table 3). The time‐specific changes in eggs without storage (0 week, A1 and A2) are connected by broken lines and shown in other panels for visual comparison with eggs stored for 1, 2, and 3 weeks. The dotted curves are pco_2_ isopleths of which values are indicated by small numerical figures. The diagonal solid line is buffer line indicating buffer value of −16 mmol L^−1^ pH ^−1^.

In 22°C storage procedure, pH of the group without storage decreased from 7.545 ± 0.007 (*N* = 38) at 0 h (before submersion) to 7.230 ± 0.012 (N = 38) at 2 h. Blood pH then increased to 7.357 ± 0.014 (N = 38) at 6 h and 7.387 ± 0.006 (N = 36) at 24 h of submersion, accompanied by changes in [HCO_3_^−^] of 29.1 ± 0.4, 28.6 ± 0.6, 35.2 ± 0.7 and 33.0 ± 0.6 mmol L^−1^ at 0, 2, 6, and 24 h, respectively (Fig. 6A1). Concomitantly, pco_2_ of 33.9 ± 0.9 at 0 h increased to 70.8 ± 1.1 mmHg at 2 h and decreased to 64.3 ± 1.4 at 6 h and 56.2 ± 1.3 mmHg at 24 h. Preincubation egg storage did not affect pH at all times of submersion, for example, pH at 0 h resulted in 7.545 ± 0.006 (N = 34), 7.551 ± 0.014 (N = 10), and 7.54 (N =1) for 1, 2, and 3 weeks storage procedures, respectively (Fig. 6B1, C1, D1) (*P* = 0.901). The difference of pH between 0, 1, and 2 weeks storage procedures was also nonsignificant at 2 h (*P* = 1), 6 h (*P* = 0.980), and 24 h (*P* = 0.338). However, [HCO_3_^−^] (and also pco_2_) was affected by storage and resulted in low values in 1 and 2 weeks storage groups compared with the nonstorage control group, for example, [HCO_3_^−^] at 0 h was 27.3 ± 0.4, 25.6 ± 0.9, and 21 mmol L^−1^ for 1, 2, and 3 week storage procedures (Fig. 6B1, C1, D1) (*P* < 0.001). At other submersion times, the difference of [HCO_3_^−^] between 0, 1, and 2 weeks storage procedures was also significant (*P* = 0.045, 0.034 and <0.001 for 2, 6, and 24 h of submersion, respectively).

In 15°C storage, pH of nonstorage group was 7.541 ± 0.007 (*N* = 37) at 0 h and decreased to 7.249 ± 0.013 (N = 37) at 2 h. Blood pH subsequently increased to 7.367 ± 0.009 (N = 37) at 6 h and 7.386 ± 0.006 (N = 38) at 24 h, accompanying changes in [HCO_3_^−^] of 27.7 ± 0.4, 28.0 ± 0.5, 34.6 ± 0.6, and 32.2 ± 0.5 mmol L^−1^ at 0, 2, 6, and 24 h, respectively (Fig. 6A2). Blood pH at 0 h increased with increasing storage, that is, 7.560 ± 0.007 (N = 34), 7.572 ± 0.008 (N = 26), and 7.589 ± 0.011 (N = 16) for 1, 2, and 3 weeks storage procedures, respectively (*P* < 0.001) (Fig. 6B2, C2, D2). However, the increase in pH due to preincubation egg storage was nonsignificant at 2 and 6 h (*P* = 0.874 and 0.266) and only significant at 24 h (*P* < 0.001). [HCO_3_^−^] (and also pco_2_) was affected by storage and resulted in low values in 1, 2, and 3 weeks storage procedures compared with nonstorage control, for example, [HCO_3_^−^] at 0 h was 26.8 ± 0.4, 25.6 ± 0.7, and 24.8 ± 0.6 mmol L^−1^ for 1, 2, and 3 week storage groups (Fig. 6B2, C2, D2) (*P* = 0.001). At other submersion times, the difference of [HCO_3_^−^] between control, 1, 2, and 3 weeks storage procedures was also significant (*P* = 0.004, 0.005, and 0.003 for 2, 6, and 24 h of submersion, respectively).

## Discussion

### Effects of prolonged preincubation egg storage

#### Viability and size of d15 embryos

Fertile eggs can be stored for several days without a major loss in hatchability if kept below the temperature required to initiate blastoderm development or maintain dormancy of embryos (i.e., so‐called “physiological zero”) (Butler 1991; Wilson 1991). The physiological zero for chicken eggs is ~25–27°C (Funk and Biellier 1944; Lundy 1969; Butler 1991; Wilson 1991). If the storage period is short (a few days), a higher storage temperature is recommended for maximum hatching success (Mayes and Takeballi 1984). Suggested temperature for storage less than 4 days is 18–28°C and the optimal temperature drops to 15–16°C for 4–7 days and 11–12°C for more than 7 days storage (Butler 1991). Similar interactions between storage temperature and time are reported, that is, 18–30°C for less than 3 days, 16–17°C for 3–7 days, and 10–12°C for more than 7 days storage (Wilson 1991). In the present study, preincubation egg storage did not affect viability of d15 embryos when eggs were stored for 1 week at both 22 and 15°C, but the viability was impaired significantly with storage duration beyond 2 weeks, particularly in 22°C storage procedure (Fig. 1A1, A2; Table 2). Water loss from eggs during storage was higher at 22°C storage temperature compared with 15°C (Fig. 1C1, C2). However, the impaired viability was not attributable to water loss from eggs, that is, storage humidity. Even if water loss was restricted or augmented by changing environmental storage humidity, the large decrease in viability of embryos occurred after 2 weeks storage at 22°C as also occurred in eggs with average water loss (Fig. 1A1, C1). Consequently, water loss from eggs linearly increased with preincubation egg storage duration, but the viability decreased irregularly and was not related to the increase or decrease in water loss.

Preincubation egg storage impaired body size of d15 embryos in eggs stored at both 22 and 15°C, that is, embryos resulted in midget due to preincubation egg storage. Mean BM of embryos decreased at storage durations of 1 and 2 weeks, particularly in eggs stored at 15°C compared with 22°C. Although maintaining the same viability, BM of embryos in eggs stored at 15°C was smaller than that stored at 22°C (Fig. 2). Consequently, although the viability of embryos was better in eggs stored at 15°C than at 22°C, the detriment to body size of embryos due to preincubation egg storage was larger in eggs stored at 15°C compared with eggs stored at 22°C.

The eggs in replicates 5 and 6 were siblings from the same flock of hens aged 27 and 31 weeks old (Table 1). Accordingly, the mean egg mass and BM of embryos in replicate 5 eggs were smaller than those of replicate 6 eggs at 0 week storage. In these small eggs of replicate 5, BM reduction due to 1 and 2 weeks storage at 22°C was larger compared with BM reduction due to 15°C storage. This difference was not seen in replicate 6, but rather BM of embryos in eggs stored for 1 week at 22°C was greater than that of embryos stored at 15°C, which was opposite to eggs in replicate 5. Similarly, in replicate 4 eggs laid by 52 weeks old hens, BM of embryos in eggs stored for 1 week at 22°C was greater than that stored at 15°C, that is, the response was opposite between eggs laid by young and advanced aged hens. Thus, in summary, the detrimental storage effect on body size of embryos may be related to the age of the hens.

#### Responses of body mass, osmolality, and lactate concentration to partial submersion

Water submersion of eggs produces various degrees of hypercapnic hypoxia for embryos, depending on the degree of submersion. Complete submersion or partial submersion with air cell at the water's surface is fatal in ~1–2 h for d15 embryos responding with reversible and dynamic changes in acid–base and hematological respiratory variables (Andrewartha et al. 2014). In contrast, half submersion with air cell down produces moderate hypercapnic hypoxia for embryos to survive for not less than 24 h, which is deemed to be long enough for production and comparison of responses. During 24 h of progressive intrinsic hypercapnic hypoxia, d15 embryos were dehydrated reducing BM as occurred in a 24 h moderate hypoxic (15% O_2_) or hypercapnic hypoxic (5% CO_2_, 15% O_2_) environment (Burggren et al. 2012). The dehydration of BM did not occur in complete submersion (Andrewartha et al. 2014), suggesting the response of water balance needs some long period, just as occurred in response to moderate intrinsic or extrinsic hypoxia or hypercapnic hypoxia exposure. These dehydration responses of BM occurred similarly during 24 h of partial submersion in eggs stored at both different temperatures, but were altered with storage duration and differently between the two storage temperatures (Fig. 3A1, A2; Table 4). A similar response of Osm occurred during submersion time in eggs stored at different temperatures, but was not altered with storage duration. However, the magnitude of Osm was affected by storage temperatures. As a result, changes in Osm during partial submersion were not related to changes in BM. In complete submersion, considerably larger increases in Osm occurred with no effects on BM, either (Andrewartha et al. 2014).

Control values of [La^−^] before submersion were low (~0.9 mmol L^−1^) and identical at all storage durations, which were the same as previously reported [La^−^] (Burggren et al. 2012; Tazawa et al. 2012; Andrewartha et al. 2014). While hypercapnia (5% CO_2_, 20% O_2_) or moderate hypercapnic hypoxia (5% CO_2_, 15% O_2_) decreases [La^−^] in chicken embryos, pure moderate hypoxia (0% CO_2_, 15% O_2_) lasting 24 h results in O_2_ debt and induction of anaerobic respiration, increasing [La^−^] (Burggren et al. 2012). However, in moderate hypoxia the increase in [La^−^] accounts for only partial decrease in [HCO_3_^−^] in metabolic acidosis produced. Severe extrinsic hypoxia (10% O_2_) with or without hypercapnia (5% CO_2_) or severe intrinsic hypercapnic hypoxia produced by complete or nearly complete submersion increases [La^−^] concurrent with decrease in [HCO_3_^−^] (Tazawa et al. 2012; Andrewartha et al. 2014). Similarly, in the present partial submersion experiment the responses of [La^−^] occurred concurrently with changes in [HCO_3_^−^] in eggs stored at both temperatures and without influences of storage duration. However, preincubation storage temperatures did alter the magnitude of the response.

#### Regulation of hematological respiratory variables

The magnitude of Hct, [RBC], and [Hb] before submersion (at 0 h) was decreased as preincubation egg storage duration was extended ≥2 weeks in eggs stored at 15°C, indicating that these variables were impaired by prolonged 15°C preincubation egg storage compared with 22°C preincubation storage.

In a 24‐h moderate hypoxia (15% O_2_) or hypercapnic hypoxia (5% CO_2_, 15% O_2_) exposure in d15 embryos, the increase in Hct was attributed to MCV only during 2–6 h and later to increase in both MCV and [RBC] at 24 h (Burggren et al. 2012; Mueller et al. 2013). In severe hypoxia (10% O_2_) or hypercapnic hypoxia (5% CO_2_, 10% O_2_), Hct was increased by increase in MCV alone with [RBC] remaining unchanged during short living period of 90–120 min (Tazawa et al. 2012; Kole et al. 2015). Contrastively, both MCV and [RBC] increased in response to complete or nearly complete submersion (Andrewartha et al. 2014; Kole et al. 2015). These previous studies on interactions of Hct, [RBC], and MCV responses were different from the present results, suggesting that interactive responses of hematological respiratory variables are unique individually among extrinsic and intrinsic hypoxia or hypercapnic hypoxia, depending on their magnitude and operational durations. Importantly, these time‐specific responses are affected by preincubation egg storage according to storage temperatures and storage durations (Figs. 4, 5), for example, regulation of Hct, [RBC], and [Hb] in response to progressive hypercapnia and hypoxia was impaired by 15°C preincubation egg storage with shorter storage duration compared with 22°C storage. In eggs stored for 3 weeks at 15°C with a higher viability than at 22°C, responses of Hct, [RBC], and [Hb] to submersion became totally different from responses of nonstorage eggs (Figs. 1A1, A2, 4A2, B2, 5D2). Consequently, physiological regulation of hematological respiratory variables is modified by preincubation egg storage according to storage temperatures and storage durations.

#### Regulation of acid–base balance

Day 15 embryos exposed to extrinsic hypercapnic hypoxia (5% CO_2_, 15% O_2_) showed a noncompensatory respiratory acidosis along the buffer line after 2 h. Subsequently, it has been shown that a partial compensatory metabolic alkalosis occurs with increase in [HCO_3_^−^] along the ~45 mmHg pco_2_ isopleth at 6 h (Mueller et al. 2013). Partial metabolic compensation in 15% O_2_ could not be preserved after 24 h and pH lowered with decrease in [HCO_3_^−^] close to noncompensatory value (Burggren et al. 2012; Mueller et al. 2013). In the present study, partial submersion of eggs into water produced progressive hypercapnia and hypoxia and accordingly embryos suffered respiratory acidosis along the buffer line. Later, additional metabolic acidosis occurred due to production of lactate in anaerobic glycolysis along the ~65–70 mmHg pco_2_ isopleth. Consequently, after 2 h of submersion, the acid–base status resulted in respiratory acidosis combined with metabolic acidosis (Fig. 6A1, A2). Subsequently, the acid–base balance was partially compensated by metabolic alkalosis with an increase in [HCO_3_^−^] after 6 h and further by respiratory alkalosis with a decrease in pco_2_ in control embryos of both 22 and 15°C preincubation storage procedures. This time course response of acid–base balance during submersion was altered by preincubation egg storage with progressive downward shift occurring with storage durations (Fig. 6A1, B1, C1 in 22°C storage procedure and Fig. 6A2, B2, C2, D2 in 15°C storage procedure). The storage temperature also caused different responses between embryos in eggs stored at 22 and 15°C. It should be noted that preincubation egg storage also modifies acid–base balance of embryos according to storage temperatures and storage durations.

### Egg storage and avian natural history

Preincubation egg storage at a low temperature is favorable for preserving viability of embryos compared with a higher storage temperature when egg storage is prolonged. Yet, lower storage temperatures deteriorate size of embryos and development of their physiological variables and functions compared with embryos in eggs stored at a higher temperature. In this context, it is interesting to consider the egg laying and nesting behavior of nondomesticated birds. Many avian species lay multiple eggs over a relatively extended period of time and, only when the last egg of the clutch is laid, begin actual incubation. This raises the possibility that eggs laid early experience a potentially far greater range of ambient temperatures and humidity prior to their eventual incubation compared to the eggs laid last, which are immediately incubated by the hen. For example, the altricial Zebra finch (*Taenropygia gurtata*) laying ≥5 eggs in a clutch starts incubation on the day when the fourth egg is laid, or the day of the last egg laid for smaller clutches (Zann and Rossetto 1991). In wild ostriches (*Struthio camelus*), several females lay more eggs than can be incubated in a communal nest. The surplus eggs frequently end up unattended during preincubation period, where they may be exposed temperatures as high as ~40°C (Bertram and Burger 1981; Kimwele and Graves 2003). Although ostriches frequently shade their eggs to prevent overheating in the hot, arid environments they inhabit, eggs laid early on or surplus eggs are exposed to such harsh environments for 2.5–3 weeks before incubation starts (Bertram and Burger 1981; Wilson et al. 1997). As another example, the Northern Bobwhite quail lays 1 egg/day for ~12–14 days and only then starts incubation ≤7 days after all laying is complete, indicating that eggs are potentially exposed to diurnally fluctuating ambient temperatures for 15–21 days before incubation (Reyna and Burggren 2012). The consequences of, and adaptations to, these preincubation exposures are unknown, but could very well induce intragenerational or even intergenerational epigenetic effects that manifest as developmental phenotypic plasticity.

### Avian egg storage, developmental plasticity, and epigenetics

Unknown is whether the detrimental effects of storage on physiological functions determined in the present study persist beyond the perinatal period into adulthood. Plasticity in development not only allows for early development to be diverted from the normal developmental trajectory, but also allows for abnormal development to be mitigated by subsequent developmental alterations, resulting in a potentially normal adult phenotype (Burggren 1998; Burggren and Reyna 2011; Burggren et al. 2014). An informative example of this is so‐called “catch‐up” or “compensatory” growth evident in a variety of animals, in which nutritionally challenged, low body mass neonates are able to move onto normal growth curves as a result of modified phenotypes as juveniles for nutrient absorption and incorporation (Won and Borski 2013; Gat‐Yablonski and Phillip 2015). In this regard, the avian egg is an excellent model for exploring nutrition‐related growth, in part, because nutritional state is related to albumin and yolk amounts in the egg (nutrient provisioning), which can be experimentally selected for either by experimental modification (Finkler et al. 1998; Nelson et al. 2010) or by selection of larger or smaller eggs (Flores‐Santin and Burggren 2015).

The epigenetic inheritance of a modified phenotype as a result of egg storage has not, to our knowledge, been examined in bird eggs. However, the intragenerational (as opposed to transgenerational) epigenetic effects of dynamic incubation temperatures or hypercapnia during incubation and the phenotypic modification they produce in the adults have been explored in the chicken and quail (De Smit et al. 2006; Shinder et al. 2011; Flores‐Santin and Burggren 2015). An investigation of the actual inheritance by the F_1_ generation of a modified phenotype as a result of conditions for egg storage and subsequent incubation is highly warranted.

### Perspectives and significance

The present study has confirmed that adjustments in physiological performance result from preincubation egg storage conditions experienced by the very earliest stages of avian embryos. While the poultry industry has long‐appreciated survival phenomena associated with storage effects, our study is the first to show that there can be significant modifications in such basic physiological processes such as acid–base balance and regulation of hematological variables. As such, early avian embryos and their response to storage may prove to be a tractable model for studying physiological effects of embryonic programming.

## Conflict of Interest

None declared.
